# Feasibility of a Short-Stay Lumboperitoneal Shunt Pathway Based on Perioperative Optimization and Individualized Discharge Decision-Making: A Pilot Before–After Study

**DOI:** 10.3390/jpm16040223

**Published:** 2026-04-17

**Authors:** Tatsuya Tanaka, Eiichi Suehiro, Anh Tran Hue, Ryosuke Doi, Shunsuke Hatakenaka, Junpei Kato, Tomihiro Wakamiya, Kimihiro Nakahara, Takashi Agari, Masahiro Indo, Takashi Sugawara, Hiroshi Itokawa, Kazuaki Shimoji, Keisuke Onoda, Akira Matsuno

**Affiliations:** Department of Neurosurgery, International University of Health and Welfare Narita Hospital, 852 Hatakeda, Narita 286-8520, Chiba, Japan; esuehiro@iuhw.ac.jp (E.S.); atranhue98@gmail.com (A.T.H.); k-nakahara@ihwg.jp (K.N.);

**Keywords:** lumboperitoneal shunt, idiopathic normal pressure hydrocephalus, personalized medicine, perioperative optimization, short-stay hospitalization, enhanced recovery, shunt valve management, feasibility study

## Abstract

**Background**: Lumboperitoneal (LP) shunt surgery is an established treatment for idiopathic normal pressure hydrocephalus (iNPH). In Japan, patients undergoing LP shunt surgery are often hospitalized for several days to more than one week after surgery, even in uncomplicated cases, reflecting concerns regarding early complications, cerebrospinal fluid overdrainage, and discharge readiness in older adults. This study evaluated the feasibility and short-term safety of a perioperative optimization pathway for planned short-stay hospitalization after LP shunt surgery. **Methods**: This single-center retrospective before-and-after cohort study included 15 consecutive patients who underwent elective LP shunt surgery. Six patients were managed using a conventional hospitalization pathway, whereas nine patients were treated under a short-stay pathway targeting discharge after one postoperative night. Key perioperative modifications included a uniform higher initial programmable valve pressure (level 7), structured discharge education, scheduled postoperative analgesia, waterproof wound sealing permitting early showering, and early outpatient follow-up with head computed tomography for staged valve pressure adjustment. The primary outcome was 30-day safety, defined as readmission, reoperation, or major postoperative complications. **Results**: Baseline characteristics were generally comparable between groups, although the short-stay group was slightly older and had more frequent antithrombotic therapy. Mean hospital length of stay was shorter in the short-stay group than in the conventional group (3.7 ± 2.0 vs. 9.7 ± 0.8 days; median, 3 vs. 9.5 days). Orthostatic headache requiring valve adjustment occurred in three conventional cases but in none of the short-stay patients. No patients in the short-stay group required readmission or reoperation within 30 days. **Conclusions**: In this pilot before-and-after study, a short-stay LP shunt pathway incorporating perioperative optimization and individualized discharge decision-making was feasible and was not associated with an apparent increase in early adverse events. These findings should be interpreted as exploratory and may support further evaluation of short-stay management strategies for selected patients undergoing LP shunt surgery in Japan.

## 1. Introduction

Lumboperitoneal (LP) and ventriculoperitoneal (VP) shunt surgeries are established treatments for idiopathic normal pressure hydrocephalus (iNPH), a disorder that predominantly affects older adults and is expected to become increasingly common as populations age worldwide [1,2,3]. Because shunt surgery can improve gait disturbance, cognitive decline, and urinary dysfunction in appropriately selected patients, optimization of perioperative care has become increasingly important in this frail and expanding patient population [1,2,4].

In Japan, patients undergoing LP or VP shunt surgery are often hospitalized for several days to more than one week after surgery, even in uncomplicated cases. This practice reflects concerns regarding early postoperative complications, cerebrospinal fluid (CSF) overdrainage, mobilization difficulties, and discharge readiness in older adults [4,5,6]. At the same time, prolonged hospitalization itself may also be associated with adverse consequences in elderly patients, including physical deconditioning, delirium, falls, and reduced independence [7,8]. These considerations raise an important clinical question: whether postoperative hospitalization after LP shunt surgery can be safely shortened in selected patients through optimized perioperative management.

In recent years, enhanced recovery after surgery (ERAS) concepts have increasingly been introduced into neurosurgical practice [6,9]. In the field of hydrocephalus surgery, previous studies—particularly those involving ventriculoperitoneal (VP) shunts—have suggested that standardized care pathways and early discharge strategies may reduce hospital length of stay without an apparent increase in readmission or complication rates [6]. These reports indicate that postoperative recovery after shunt surgery may be influenced not only by the procedure itself but also by perioperative care design. However, data specifically addressing short-stay strategies after LP shunt surgery remain limited.

LP shunting has several clinical features that may influence discharge timing. In particular, concern regarding early CSF overdrainage, orthostatic headache, and subdural collections has traditionally supported cautious postoperative observation [1,4]. In conventional practice at our institution, initial programmable valve pressure was determined according to the Miyake formula based on sex, body weight, and height [10]. Although this strategy is physiologically reasonable, lower initial valve settings in some patients may contribute to early overdrainage-related symptoms requiring inpatient observation or valve adjustment.

Importantly, discharge readiness after LP shunt surgery depends on more than valve pressure alone. In older adults with iNPH, postoperative stability may also be influenced by pain control, wound management, mobilization, oral intake, caregiver support, and timely outpatient follow-up. Despite this, most previous reports have primarily focused on standardized institutional protocols or discharge timing itself, rather than on a broader perioperative framework integrating multiple clinically relevant factors.

Over the past several years, our institution has progressively refined LP shunt surgery with the aim of improving technical stability and reducing shunt-related complications, including the use of a one-piece shunt system, fluoroscopic guidance, and procedural modifications to minimize valve-related or catheter-related failure [11,12]. Building upon this technical foundation, we introduced a revised perioperative management pathway intended to facilitate planned short-stay hospitalization. This pathway incorporated a conservative initial valve pressure strategy, scheduled postoperative analgesia, waterproof wound sealing to support early activity, structured discharge education for patients and caregivers, and early outpatient reassessment with head computed tomography (CT) for staged valve pressure adjustment.

In this study, the concept of personalized perioperative optimization refers not to a single individualized intervention, but to a coordinated perioperative framework in which discharge decisions were made according to patient-specific postoperative stability, functional recovery, symptom burden, and home support readiness. To our knowledge, limited data are available regarding short-stay LP shunt management evaluated within such an integrated perioperative framework.

Therefore, the aim of this pilot before-and-after study was to evaluate the feasibility and short-term safety of a short-stay LP shunt pathway based on perioperative optimization and individualized discharge decision-making, compared with conventional hospitalization.

## 2. Materials and Methods

### 2.1. Study Design and Ethical Approval

This was a single-center retrospective before-and-after cohort study conducted at the International University of Health and Welfare Narita Hospital, Japan.

The study was conducted under institutional ethical approval allowing retrospective use of clinical data (Approval No. 23-Nr-010). The present short-stay pathway itself was not a prospectively approved interventional protocol; rather, clinical data were retrospectively analyzed after implementation of the modified perioperative strategy. Written informed consent for the use of clinical data was obtained from all patients.

### 2.2. Patient Population

We retrospectively reviewed consecutive patients who underwent LP shunt surgery using the one-piece method between January 2023 and December 2025 [11].

Eligible patients were those admitted from home for the primary purpose of elective LP shunt surgery for iNPH.

#### Exclusion Criteria

The following patients were excluded:

Patients who underwent LP shunt surgery during hospitalization for other acute conditions (e.g., cerebellar infarction or meningitis).

Patients transferred from another hospital while already hospitalized and subsequently discharged back to the referring institution.

Thus, the study population consisted of patients electively admitted from home specifically for LP shunt surgery.

Patients were categorized according to treatment period:

- Conventional hospitalization group (pre-implementation period);
- Short-stay group (post-implementation period).

Group allocation was defined according to treatment period (before vs. after implementation), and patients were analyzed according to this allocation regardless of achieved length of stay.

### 2.3. Surgical Procedure: One-Piece Method [11]

All procedures were performed under general anesthesia.

The one-piece method was adopted in all cases. The technique consisted of:

- One-piece catheter assembly to eliminate connector-related mechanical failure;
- Intraoperative fluoroscopic guidance to confirm appropriate lumbar catheter positioning;
- Subfascial shallow placement of the programmable valve to enhance mechanical stability;
- Use of an inline shunt passer to prevent catheter twisting or stress.

These technical refinements were designed to reduce valve flipping, catheter migration, mechanical stress, and shunt malfunction, thereby improving postoperative stability.

### 2.4. Valve Pressure Strategy

In the conventional group, the initial programmable valve pressure was determined according to the Miyake formula, based on sex, body weight, and height [10].

In the short-stay group, the initial valve pressure was uniformly set at level 7 to reduce early cerebrospinal fluid overdrainage and orthostatic headache.

Head CT was performed approximately one week postoperatively, and gradual pressure reduction was initiated thereafter based on clinical and radiological assessment.

### 2.5. Short-Stay Pathway

The short-stay protocol targeted discharge after one postoperative night (postoperative day 1).

Discharge criteria included:

- Stable neurological findings without new deficits;
- Hemodynamic stability;
- Adequate pain control with oral medication;
- Ability to tolerate oral intake;
- Early mobilization;
- No clinically significant CSF leakage;
- No severe postoperative delirium;
- Availability of home caregiver support.

If criteria were not met, hospitalization was extended at the treating physician’s discretion.

Additional refinements implemented during the short-stay period included:

- Waterproof wound sealing permitting showering from postoperative day 1;
- Scheduled oral analgesics for five days;
- Provision of structured discharge education materials;
- Immediate initiation of home-based nursing services;
- Outpatient follow-up at approximately one week.

A structured discharge instruction leaflet outlining wound care, warning symptoms, activity guidance, and emergency contact information was provided to all patients and caregivers at discharge (File S1).

No patients required routine postoperative ICU management. No concomitant major surgical procedures were performed during index admission.

### 2.6. Outcomes

#### 2.6.1. Primary Outcome

The 30-day safety, defined as:

- Unplanned readmission;
- Reoperation;
- Shunt-related complication requiring intervention;
- Surgical site infection;
- Symptomatic subdural hematoma.

#### 2.6.2. Secondary Outcomes

- Length of hospital stay;
- Orthostatic headache;
- Postoperative delirium;
- Intraoperative complications.

Late complications beyond 30 days were recorded descriptively.

### 2.7. Statistical Analysis

Given the pilot nature of the study and small sample size (*n* = 15), analyses were primarily descriptive.

Continuous variables are presented as mean ± standard deviation or median values. Categorical variables are presented as counts and percentages.

Exploratory comparisons between groups were performed using the Mann–Whitney U test for continuous variables and Fisher’s exact test for categorical variables. However, the study was not powered to detect statistically significant differences.

## 3. Results

### 3.1. Study Population

Between January 2023 and December 2025, 17 patients underwent LP shunt surgery using the one-piece method. Two patients were excluded: one who was transferred from another institution during hospitalization and discharged back to the referring hospital, and one who underwent LP shunt placement during admission for cerebellar infarction and meningitis.

A total of 15 patients admitted electively from home for LP shunt surgery were included in the analysis.

Six patients were treated during the conventional hospitalization period, and nine patients were treated after implementation of the short-stay pathway targeting discharge after one postoperative night.

### 3.2. Baseline Characteristics

Baseline characteristics are summarized in Table 1.

The short-stay group was slightly older (78.9 ± 4.2 years) than the conventional group (73.3 ± 10.7 years). Mean MMSE scores were identical between groups (20.3 in both groups). The prevalence of diabetes was similar (17% vs. 11%). Antithrombotic therapy was numerically more frequent in the short-stay group (56%) compared with the conventional group (17%). Smoking history, alcohol use, BMI, and sex distribution were comparable.

Overall, no clinically meaningful baseline differences suggesting lower perioperative risk were observed in the short-stay group.

### 3.3. Perioperative Variables

Perioperative variables are presented in Table 2.

All procedures were performed under general anesthesia using the one-piece method with intraoperative fluoroscopic guidance.

Median operative time was similar between groups (40 min in the conventional group vs. 45 min in the short-stay group). The longer mean operative time in the short-stay group (61.3 ± 36.1 min) was influenced by one case of intraoperative bowel injury. Estimated blood loss was minimal in both groups.

A distinct difference was observed in the initial programmable valve pressure strategy. In the conventional group, the mean initial pressure was 5.5 ± 1.5, determined according to the Miyake formula. In contrast, all patients in the short-stay group were uniformly set at pressure level 7.

Orthostatic headache requiring valve adjustment occurred in three patients (50%) in the conventional group but in none of the short-stay patients.

### 3.4. Postoperative Outcomes

Postoperative outcomes are summarized in Table 3.

#### 3.4.1. Length of Hospital Stay

The mean length of stay was 9.7 ± 0.8 days in the conventional group and 3.7 ± 2.0 days in the short-stay group. The median length of stay was 9.5 days versus 3 days, respectively.

One patient in the short-stay group experienced an intraoperative bowel injury that was immediately recognized and repaired during the same procedure. Although discharge had initially been planned after one postoperative night, hospitalization was extended for observation (total stay: nine days). The patient recovered without additional morbidity and did not require reoperation.

All other patients in the short-stay group were discharged after one postoperative night as planned.

#### 3.4.2. The 30-Day Safety Outcomes

Within 30 days postoperatively:

No patients in the short-stay group required unplanned readmission.

No patients in the short-stay group required reoperation.

No postoperative delirium was documented in the short-stay group.

No symptomatic subdural hematoma or surgical site infection occurred.

In the conventional group, one patient (16.7%) was readmitted approximately two weeks after discharge due to a fall-related acute subdural hematoma. No reoperations occurred within 30 days in either group.

One patient in the conventional group developed postoperative delirium during the index admission.

#### 3.4.3. Late Complications

One patient in the conventional group experienced shunt rupture requiring lumbar catheter reconstruction 18 months after surgery. This late mechanical complication was not included in the 30-day endpoint analysis.

## 4. Discussion

### 4.1. Principal Findings

In this single-center before-and-after cohort study, we evaluated the feasibility and short-term safety of a short-stay LP shunt pathway targeting discharge after one postoperative night. In the Japanese clinical setting, postoperative hospitalization after LP shunt surgery often remains relatively prolonged, even in uncomplicated cases, because of concerns regarding early complications, CSF overdrainage, and discharge readiness in older adults. Within this context, our findings suggest that a structured short-stay pathway may be feasible in selected patients and was not associated with an apparent increase in 30-day readmissions, reoperations, or major shunt-related complications.

A notable observation was that orthostatic headache requiring early valve adjustment occurred in three patients in the conventional group but in none of the patients managed under the short-stay pathway. Although this difference should be interpreted cautiously given the small sample size and non-randomized design, it raises the possibility that perioperative optimization—particularly a more conservative initial valve pressure strategy—may have contributed to improved early postoperative stability. At the same time, the absence of early adverse events in the short-stay group should not be interpreted as definitive evidence of safety, but rather as the absence of detected complications in a small pilot cohort. Accordingly, the present findings should be regarded as exploratory and hypothesis-generating rather than confirmatory.

### 4.2. Short-Stay Strategy in the Context of Existing Literature

Early discharge protocols following VP shunt surgery have been reported to reduce length of stay without increasing postoperative complications or healthcare utilization [6]. However, evidence specific to LP shunt surgery remains limited [5]. This distinction is important because LP shunting presents several technical and physiological considerations that may influence postoperative management, particularly with respect to CSF overdrainage, orthostatic headache, and subdural collections [1,4].

In conventional practice at our institution, the initial programmable valve pressure was determined using the Miyake formula, based on sex, body weight, and height [10]. Although this approach is physiologically reasonable and widely accepted, lower initial settings in some patients may be associated with early overdrainage-related symptoms requiring observation or valve adjustment. In contrast, during the short-stay period, all patients were initially managed with a uniform pressure setting of level 7. In the present cohort, this strategy was associated with the absence of early orthostatic headache requiring valve adjustment.

This observation should not be interpreted as proof that the valve strategy alone accounted for the differences observed between groups. Because this study used a retrospective before-and-after design, multiple factors may have contributed, including temporal changes in practice, increasing familiarity with perioperative management, and unmeasured patient-related differences. Nevertheless, the findings suggest that conservative early valve management may be a clinically relevant component of short-stay LP shunt care and warrants further study.

Importantly, the present study differs from prior early discharge reports in that it did not evaluate discharge timing as an isolated institutional protocol. Rather, it examined short-stay management within a broader perioperative framework that incorporated valve management, discharge preparation, postoperative symptom control, and structured follow-up. In this sense, the present study contributes to the limited literature by suggesting that short-stay LP shunt care may be better understood as a perioperative systems issue rather than simply a shortened observation period.

### 4.3. Interpretation of Complications

One intraoperative bowel injury occurred in the short-stay group. The injury was immediately recognized and repaired during the same procedure, hospitalization was extended for observation, and the patient recovered without additional morbidity or need for reoperation. This event represents a recognized but uncommon complication of LP shunt placement and does not appear to have been directly related to the discharge pathway itself. Importantly, the pathway allowed sufficient flexibility to extend hospitalization when clinically indicated, rather than enforcing discharge according to a rigid timeline.

In the conventional group, one patient was readmitted approximately two weeks after discharge because of a fall-related acute subdural hematoma. Although this event occurred after a longer index hospitalization, it highlights an important clinical point: prolonged hospitalization does not necessarily eliminate post-discharge risk in elderly patients with iNPH. In this population, postoperative vulnerability may be influenced not only by shunt-related factors, but also by frailty, gait instability, cognitive impairment, and reduced physiological reserve. Therefore, discharge safety may depend less on hospitalization duration alone than on the quality of discharge preparation, caregiver readiness, and post-discharge support.

Taken together, these events underscore that complications after LP shunt surgery should be interpreted within a broader geriatric and perioperative context. A short-stay strategy should not be viewed as a universal acceleration of discharge, but rather as a framework requiring clinical flexibility, structured readiness assessment, and appropriate follow-up.

### 4.4. Personalized Perioperative Optimization

A distinguishing feature of the present study is that early discharge was not approached simply by shortening the observation period, but through coordinated restructuring of perioperative management. The short-stay pathway incorporated a conservative initial valve pressure strategy to reduce the likelihood of early CSF overdrainage, water-proof wound sealing that permitted showering from postoperative day 1 and thereby supported early mobilization, scheduled postoperative analgesia to improve comfort and function, and structured discharge education to improve post-discharge safety awareness among patients and caregivers. In addition, outpatient reassessment—including imaging—was systematically performed approximately one week after surgery to guide staged valve pressure adjustment.

In this study, the concept of personalized perioperative optimization should be understood in a practical clinical sense. It did not refer to a single biologically individualized intervention, but rather to a coordinated framework in which discharge decisions were made according to patient-specific postoperative stability, symptom burden, oral intake, mobility, and availability of home support. This distinction is important, because the novelty of the present study lies less in any one intervention than in the integration of multiple perioperative elements into a clinically individualized discharge process.

Accordingly, the present findings should not be interpreted as evidence that one specific modification “caused” shorter hospitalization. Rather, they suggest that short-stay management after LP shunt surgery may be feasible when discharge timing is embedded within a broader perioperative optimization strategy tailored to postoperative recovery status.

### 4.5. Foundational Surgical Optimization

The feasibility of short-stay hospitalization in this study was built upon prior institutional refinement of shunt stabilization techniques. Use of the one-piece method, subfascial shallow valve placement, and intraoperative fluoroscopic guidance were consistently applied throughout the study period [11,12]. These technical standardizations were intended to reduce mechanical failure, catheter migration, and valve-related complications, thereby improving procedural reliability.

This technical background is important when interpreting the present findings. Short-stay hospitalization is unlikely to be safely implemented in isolation if the underlying surgical system remains vulnerable to avoidable technical complications. In other words, discharge strategy and operative reliability are closely linked. The present study therefore supports the concept that perioperative pathway development should be built upon a stable technical foundation.

This stepwise evolution—from complication prevention to perioperative optimization and ultimately to individualized discharge planning—may be particularly relevant in LP shunt surgery, where relatively small technical or valve-related issues can substantially influence early postoperative symptoms and discharge readiness.

### 4.6. Anesthesia Considerations

Although LP shunt surgery has been reported to be feasible under local or spinal anesthesia in selected patients [5,13], all procedures in the present study were performed under general anesthesia. This reflects the standard practice at our institution during the study period. Despite this, short-stay hospitalization was still achieved in most patients managed under the revised pathway.

This observation suggests that anesthesia modality alone may not be the primary determinant of discharge timing. Rather, discharge readiness after LP shunt surgery may depend more strongly on the overall perioperative framework, including symptom control, mobilization, wound management, valve strategy, and structured follow-up. At the same time, because anesthesia type was not varied in this study, no comparative conclusions can be drawn regarding its influence on postoperative recovery or feasibility of early discharge.

### 4.7. Clinical Implications

The present findings suggest that routine prolonged hospitalization after LP shunt surgery may not be universally necessary in all patients. In selected individuals, discharge after one postoperative night may be feasible when supported by structured perioperative management and individualized discharge decision-making. This perspective may be particularly relevant in Japan, where hospitalization after shunt surgery often remains relatively long compared with the procedural invasiveness of the operation itself.

The potential implications extend beyond length of stay alone. In older adults with iNPH, avoiding unnecessary hospitalization may help reduce exposure to immobility, sleep disruption, delirium risk, and functional decline [7,8]. In addition, from a healthcare systems perspective, short-stay pathways may contribute to more efficient bed utilization in aging societies facing increasing procedural demand and resource constraints.

At the same time, the present findings should not be interpreted as supporting uniform next-day discharge for all patients undergoing LP shunt surgery. Rather, they support a more selective and clinically individualized approach in which hospitalization duration is determined by recovery status and support readiness rather than by routine institutional convention alone.

### 4.8. Limitations

Several limitations must be acknowledged. First, the sample size was small, particularly in the short-stay group, limiting statistical power and precluding definitive conclusions regarding rare complications or subgroup effects. Importantly, the absence of major early complications in this cohort should not be interpreted as proof of safety, but rather as an absence of detected events within a limited sample.

Second, the before-and-after design introduces potential confounding factors, including temporal changes in perioperative practice, increasing clinical familiarity with the pathway, and other unmeasured differences between treatment periods. Therefore, the observed group differences cannot be attributed to any single intervention.

Third, because the short-stay pathway consisted of multiple coordinated changes—including valve strategy, discharge preparation, symptom management, and follow-up structure—the relative contribution of each component cannot be determined from the present study.

Fourth, functional outcomes, patient-reported recovery, caregiver burden, and quality-of-life measures were not systematically assessed, although these may be particularly important when evaluating hospitalization strategies in older adults with iNPH. In this regard, future studies should not focus solely on hospital length of stay or early adverse events, but also incorporate patient-centered measures that better capture the true value and acceptability of short-stay care.

Finally, this study was conducted at a single center with highly standardized operative techniques and a specific clinical workflow in Japan, which may limit generalizability to other institutions or healthcare systems. Accordingly, the present findings should be interpreted as preliminary feasibility data and hypothesis-generating observations rather than definitive evidence of superiority or non-inferiority.

## 5. Conclusions

In this pilot before-and-after study, a short-stay LP shunt pathway incorporating perioperative optimization and individualized discharge decision-making was feasible and was not associated with an apparent increase in early adverse events. These findings suggest that, in selected patients, discharge after one postoperative night may be achievable within a structured perioperative framework. However, because of the small sample size and observational design, the present results should be interpreted as exploratory. Further studies are needed to clarify the safety, reproducibility, and broader applicability of short-stay LP shunt management, particularly in the Japanese clinical setting.

## Figures and Tables

**Table 1:** Baseline Characteristics.

Variable	Conventional (*n* = 6)	Short-Stay (*n* = 9)
Age (years), mean ± SD	73.3 ± 10.7	78.9 ± 4.2
Female sex, *n* (%)	2 (33%)	2 (22%)
BMI (kg/m^2^), mean ± SD	23.3 ± 3.6	26.3 ± 4.8
MMSE score, mean ± SD	20.3 ± 6.9	20.3 ± 4.3
Diabetes mellitus, *n* (%)	1 (17%)	1 (11%)
Antithrombotic therapy, *n* (%)	1 (17%)	5 (56%)

**Table 2:** Perioperative Characteristics.

Variable	Conventional (*n* = 6)	Short-Stay (*n* = 9)
Operative time (min), mean ± SD	39.8 ± 4.9	61.3 ± 36.1
Operative time (min), median	40	45
Estimated blood loss (mL), mean	1.7	5.3
Initial valve pressure, mean	5.5	7.0 (uniform)
Orthostatic headache requiring adjustment	3 (50%)	0 (0%)
Intraoperative complication	0	1 (bowel injury)

**Table 3:** Postoperative Outcomes.

Outcome	Conventional (*n* = 6)	Short-Stay (*n* = 9)
Length of stay (days), mean ± SD	9.7 ± 0.8	3.7 ± 2.0
Length of stay (days), median	9.5	3
30-day readmission	1 (16.7%)	0 (0%)
30-day reoperation	0	0
Postoperative delirium	1 (16.7%)	0 (0%)
Late shunt rupture (>30 days)	1	0

## Data Availability

The data supporting the findings of this study are not publicly available due to patient privacy and ethical restrictions but are available from the corresponding author upon reasonable request.

## Supplementary Materials

The following supporting information can be downloaded at: https://www.mdpi.com/article/10.3390/jpm16040223/s1 File S1: Discharge instruction leaflet used in the short-stay LP shunt pathway, including guidance on wound care, activity, warning signs, and contact information.

## Abbreviations

The following abbreviations are used in this manuscript:

LP	Lumboperitoneal
VP	Ventriculoperitoneal
iNPH	Idiopathic Normal Pressure Hydrocephalus
CSF	Cerebrospinal Fluid
ERAS	Enhanced Recovery After Surgery
LOS	Length of Stay
CT	Computed Tomography
MMSE	Mini-Mental State Examination
BMI	Body Mass Index
SD	Standard Deviation
