# Supplementary material for: Feasibility of a Short-Stay Lumboperitoneal Shunt Pathway Based on Perioperative Optimization and Individualized Discharge Decision-Making: A Pilot Before–After Study
Source: J Pers Med. 2026 Apr 17;16(4):223. doi: 10.3390/jpm16040223 (PMC13117162; doi:10.3390/jpm16040223)
Supplement: Supplementary file 1 [file jpm-16-00223-s001.zip › jpm-4210900-supplementary.pdf]

Discharge Instructions After Lumboperitoneal (LP) Shunt Surgery

1. Introduction

This leaflet provides important instructions to help ensure a safe recovery at home after LP shunt surgery. Please review this information carefully with both the patient and caregivers.

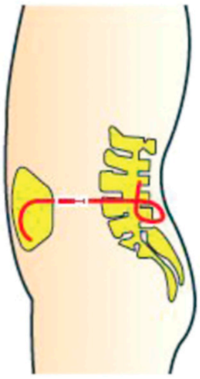

2. Wound Care and Management

- Avoid pressing, rubbing, or touching the surgical sites on the lower back and abdomen.
- Waterproof dressings are applied to the wounds, and bathing is permitted from the day of discharge.
- If the dressing becomes detached, cover the wound with a clean adhesive bandage.
- Do not scratch or rub the wound, even if itching occurs.
- Mild swelling or subcutaneous bruising may occur but usually improves over time.

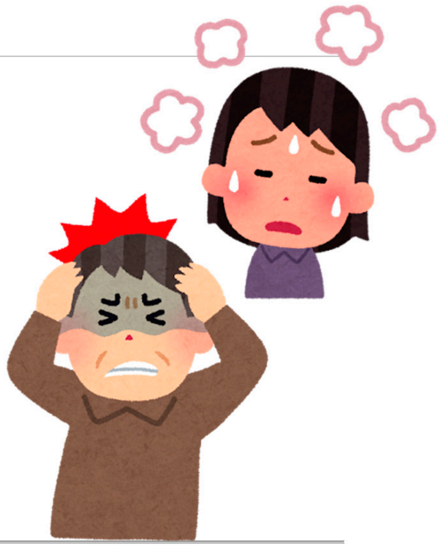

3. Symptoms Requiring Medical Attention

Please contact the hospital promptly if any of the following symptoms occur:

- Severe or persistent headache
- Repeated vomiting
- Reduced alertness or slowed responsiveness
- Fever of 38° C (100.4° F) or higher

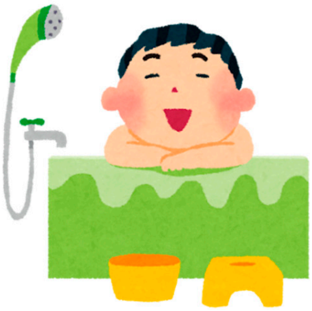

4. Daily Activity Guidelines

- Resume daily activities gradually according to your physical condition.
- Avoid strenuous physical activity until your next outpatient visit.
- Bathing and use of day-care services are permitted from the day of discharge.

5. Contact Information

Department of Neurosurgery
International University of Health and Welfare Narita Hospital
Attending physician: Tatsuya Tanaka
TEL: +81-476-35-5600 (Weekdays 9:00–17:00)
For emergencies outside these hours, please contact the emergency department.
